# Does economic policy uncertainty matter to explain connectedness within the international sovereign bond yields?

**DOI:** 10.1007/s12197-021-09554-8

**Published:** 2021-06-11

**Authors:** Noureddine Benlagha, Wael Hemrit

**Affiliations:** 1 Department of Finance and Economics, College of Business and Economics, Qatar University, P.O.X 2713, Doha, Qatar; 2 Department of Insurance and Risk Management, College of Economics and Administrative Sciences, Imam Mohamed Ibn Saud Islamic University (IMSIU), P.O. Box 5701, Riyadh, Saudi Arabia, and GEF2A Laboratory, ISG Tunis, University of Tunis, Tunisia

**Keywords:** Sovereign Bonds, G7 Countries, Economic Policy Uncertainty, Connectedness, G15, C51, C58, F65

## Abstract

This paper examines the determinants of the dynamic connectedness between sovereign bond yields in a sample of G7 countries. In addition to the common macroeconomic factors, we focus on the impact of Economic Policy Uncertainty (EPU) on the dynamic connectedness patterns between bond yields. To this end, we first examine the full-sample connectedness among the seven bond yields and examine various features of connectedness using a measure recently proposed by Diebold and Yilmaz (Int J Forecast 28(1):57-66, 2012). To examine the determinants of the dynamic connectedness, we use the panel data model to consider the dynamic net connectedness between the considered bond yields as the endogenous variable. Overall, being the transmitter or recipient of spillovers appears to have independent and different influences depending on each of the two types of sovereign bond yields. Also, the findings support the idea that EPU can create an environment likely to exacerbate the transmission of spillover shocks between two-year sovereign bond yields. Conversely, on the whole, EPU does not appear to affect the connectedness of thirty-year sovereign bond yields in various bond markets. The findings also reveal the significant impacts of real output on how shocks across countries manifest in different ways.

## Introduction

There has been growing interest in analysing spillover and dynamic connectedness across international financial markets, especially after the emergence of the US subprime mortgage and European sovereign debt crises (Meegan et al. 2018; Kim et al. 2015; Jung and Maderitsch 2014). In these studies, much attention has been placed upon how the financial crises affected dynamic spillovers among international financial markets, and their findings have suggested a significant increase in spillovers during a period of financial turmoil. However, despite the importance of sovereign bonds for institutional investment portfolios, and for individual investors, a review of the existing literature revealed few studies that have investigated the spillovers and connectedness among this particular asset class. Spillover effects are highly relevant to regulators, financial professionals and investors investing in portfolios consisting of sovereign bonds. Handler and Jankowitsch (2018) suggest that sovereign bonds represent the most directly affected financial instruments and understanding their price reactions offers significant insides, enriching the results presented for stock and option markets.

Prior studies that have identified shocks transmission between bond markets, which generally focus on the effects of the benchmark term structure of interest rates on bond risk premia, spread the first moment and assume a non-informational interaction between sovereign bond volatilities (Cepni et al. 2019; Presbitero et al. 2016a). Another branch of literature has relied exclusively on isolated studies of target counties and regions or a very small group of economies, most of which have operated under very special circumstances. Accordingly, they did not take into consideration the very serious multilateral linkage between countries. As a result, this can lead to weak predictive looseness and robustness of empirical testing. This study, therefore, sets out to assess the dynamic spillovers and connectedness among sovereign bond markets of the G7 countries (US, Canada, UK, France, Germany, Italy and Japan) over the period from January 2015 to December 2019.

As opposed to most of the previous work that focuses on the exploration of the aggregated spillovers among markets, we employ the methodology of Diebold and Yilmaz (2012) to investigate the dynamic net connectedness among the considered bond yields. To assess the sensitivity of dynamic connectedness for a specific investment horizon, we also investigate simultaneously the spillovers among bonds that take two years and thirty years to mature. In addition to attempting to measure the degree of connectedness and their sensitivity to time horizons, this paper examines how macroeconomic factors such inflation rates, the real interest rate and the economic growth influence the dynamic of net connectedness among the considered sovereign bond yields. Historically, research investigating the factors associated with dynamic spillovers between assets has focused on the standard macroeconomic variable (see, for example, Capelle-Blancard et al. 2019; Vácha et al. 2019; Costantini et al. 2014; Ghosh et al. 2013; Benlagha 2020). Unlike these studies, further to the standard macroeconomic factors, this paper pays special attention to the impact of EPU on the patterns of dynamic connectedness between the G7 sovereign bond yields.

During the last several decades, the world has become full of uncertainty as a result of financial crises, wars and the current COVID-19 pandemic. Against this background, spillovers and connectedness have increased sharply among several assets. Thus, a natural question is raised: does economic policy uncertainty (EPU) affect the dynamic connectedness between sovereign bond yields? To the best of our knowledge, this paper is the first to offer and answer to the preceding inquiry.

The need to understand and measure the effects of uncertainty on economic policy and the receiving country’s characteristics on the net connectedness across sovereign debt markets is an important topic in finance research. Decisions that rely on this understanding include whether to take advantage of arbitrage opportunities, whether to combine hedging operations or whether to share risks rather than sharing a ‘common’ sovereign bond. Understanding the determinants of connectedness can help to predict changes in Sovereign Bond Yields (SBY), which can affect government and borrowing costs and, consequently, affect the financial sector. Not only does EPU affect interest rate levels, but it also explains the level and shape of the term structure of bond yield volatilities.

Analysts and investors alike place great value in the yield spread. Investors think that EPU leads to a worsening deficit by putting bond yields under pressure in the short term and believe they can get a higher return on investment with a two-year bond than with a thirty-year bond market (Leippold and Matthys 2015). Thus, it is important to identify both near-term aspects (e.g., when the government adjusts its policy rate and regulates the issuance of government bonds) and longer-term aspects (e.g., how to implement entitlement programs). In this respect, the main objective of this study is to bridge the literature examining the impact of EPU with the literature on spillovers between sovereign bond markets at various maturities in countries around the world. First, we explore the dynamic patterns of connectedness between sovereign bond yields of the G7 countries (the US, Canada, France, the UK, Germany, Italy and Japan). We employ the methodology proposed by Diebold and Yilmaz (2009). Second, we assess the influence of EPU and several macroeconomic variables on dynamic net spillovers and net connectedness between bond yields of the selected countries. Empirically, we estimate and analyse several panel data models by regressing the net connectedness of each sovereign bond yields on macroeconomic variables affecting bond yields, namely, the inflation and interest rate along with EPU index.

The remainder of the paper is structured in the following way: Section 2 reviews the literature. Section 3 describes the data and the summary statistics. Section 4 presents the models and the estimation method. Section 5 presents and discusses the empirical results. Section 6 concludes.

## Literature review

This paper draws on various strands of the literature related to spillover and connectedness patterns among international financial assets. Considerable research has been devoted to investigating the spillovers between stock markets (for instance, Eun and Shim 1989; Hamao et al. 1990; Barclay et al. 1990); these studies advocate that the foremost stock market returns and volatilities are interconnected and demonstrated strong evidence of volatility spillovers between various developed markets. A growing body of literature followed these foundational works on spillovers and connectedness among several global stock markets (see, for instance, Chou et al. 1999; Garvey et al. 2001; Al-Deehani and Moosa 2006; Beirne et al. 2010; Horta et al. 2014; Golosnoy et al. 2015; Baruník et al. 2016; Finta and Aboura 2020; Atenga and Mougoué 2020; Weiping et al. 2020). In addition to stock markets, the studies on spillovers and interconnectedness have been extended to other markets such as commodities (Yip et al. 2017; Chevallier and Ielpo 2013), conventional currencies (Bouri et al. 2018; Bubak et al. 2011) and digital markets (Ji et al. 2019; Giudici and Pagnottoni 2019; Corbet et al. 2018).

Despite the increased utility of sovereign bond yields for investors and policymakers, few studies investigated the spillovers and connectedness among them (Ahmad et al. 2018; Piljak 2013; Antonakakis and Vergos 2013; Kim et al. 2006). De Santis and Zimic (2018) suggest that the lack of previous research studies on this topic is due first to opposing forces, such as flight-to-safety and flight-to liquidity on the one hand and fire sales on the other hand, which make it difficult to predict whether the spillovers are more likely to generate positive or negative correlation. Second, it is difficult to generate mutually exclusive sign restrictions that would properly identify a set of sovereign bond price specific shocks. Previous research has drawn different conclusions. For example, Kim et al. (2006) have examined the integration of European government bond markets using a set of GARCH models. Their findings show evidence of dynamic linkages between Eurozone bond markets with that of Germany, and there is weaker evidence outside of the Eurozone for other select European countries. Overall, their results on the linkage among the studied countries are inconclusive and failed to explain the directional spillovers between the sovereign bond markets. In another study, Antonakakis and Vergos (2013) used the VAR-based spillover index approach of Diebold and Yilmaz (2012) to assess spillovers effects between Sovereign Bond Spreads (SBS) in the Euro area during a turbulent period. Their findings show that on average, SBY spread shocks tend to increase future SBYs and are related to news announcements and policy changes. This empirical study is especially interesting because it provides a complete description of the directional spillovers among the studied markets, which earlier studies did not. However, this study is limited to sovereign bonds in the Eurozone area and did not offer any insight into the determinants of the observed differences in the directional spillovers among the studied sovereign bond markets. Fernández-Rodríguez et al. (2015) used the Diebold and Yilmaz (2014) framework on data covering the period 1999 to 2014 and find that, during the pre-crisis period, the volatility spillovers are most pronounced in the EMU sovereign bond market of central countries and peripheral countries imported credibility from them, while during the crisis peripheral countries, they are converted to the dominant transmitters. In the same way, Conefrey and Cronin (2015) find that the euro area sovereign bond crisis has moved from being driven initially by broadly-based systemic concerns to a later focus on country-specific developments.

This paper is closely related to the works of Antonakakis and Vergos (2013) and Fernández-Rodríguez et al. (2015) as we use the VAR-based spillover index approach of Diebold and Yilmaz (2012). However, our study is quite different on several points. First, the prior studies did not consider the time horizon effects on the connectedness among assets. Essentially, the maturity of bonds is an important variable that might affect the results and the dynamic patterns of connectedness between sovereign bonds. Unlike previous studies, this paper considers sovereign bonds with different maturities. Second, previous studies did not investigate the determinants of the dynamic patterns of connectedness among bond yields.

## Data and summary statistics

### Data

To explore the dynamic connectedness patterns between SBYs and their determinants, we used several datasets.

We consider monthly SBYs with maturities of two years (2YBYs) and thirty years (30YBYs) for selected developed countries (the US, Canada, the UK, France, Germany, Italy and Japan) from January 2015 to December 2019. The data for the yields of bonds was extracted from *Eikon*.

We used the specific monthly EPU Index at www.policyuncertainty.com for each country. According to Baker et al. (2013), and EPU index is a good proxy for uncertainty about the economic policy. In addition to the SBY and EPU data, we used monthly series on inflation represented by the consumer price index (CPI) and the real interest rate of each considered country. The macroeconomic dataset was extracted from DataStream.

### Summary statistics

In this section, we report and describe the main statistical features of 2YBYs and 30YBYs. For the sake of brevity, a detailed statistical analysis of other variables is reported in supplementary documents and can be made available upon request.

Table 1 reports the summary statists of the 2YBYs and 30YBYs in panel A and panel B, respectively. The table shows that, for the period under review, Italy had the highest 2YBYs, followed by France and Canada. The lowest average is detected in the Japanese bond yields. Italy also presented the highest average 30YBYs, followed by the US. The lowest average was also attributed to Japan. The unconditional volatility, measured by standard deviation, was relatively similar across all the 2YBYs, except for Japan, which was significantly low compared to others. The same result was observed for the 30YBYs. For all countries, the unconditional volatility of 2YBYs surpassed those of thirty years, except Japan, and all the studied 2YBYs series presented positive skew, except for Germany. Conversely, most of the studied 30YBYs series exhibited a negative skew. An excess of kurtosis was observed only for Italy and Japan. Finally, the Jarque-Bera test indicated a significant departure from normality for all the studied series.

**Table 1:** Summary statistics of the 2YBYs and 30YBYs

	US	CANADA	GERMANY	UK	FRANCE	ITALY	JAPAN
Panel A. 2YBYs							
Mean	2.933	3.138	2.167	3.383	2.343	3.381	0.364
Median	2.462	3.007	2.473	4.171	2.503	2.963	0.151
Maximum	7.689	8.680	6.774	8.214	7.439	12.845	3.023
Minimum	0.203	0.397	-0.921	0.075	-0.843	-0.332	-0.322
Std. Dev.	2.179	2.040	2.019	2.580	2.130	2.842	0.590
Skewness	0.333	0.574	-0.008	0.064	0.201	1.148	2.465
Kurtosis	1.650	2.433	1.762	1.474	2.091	4.116	10.163
Jarque-Bera	29.164	21.079	19.727	30.200	12.721	83.919	973.454
Probability	0.000	0.000	0.000	0.000	0.002	0.000	0.000
Panel B. 30YBYs							
Mean	4.070	3.739	3.274	3.632	3.624	4.608	1.811
Median	4.298	3.845	3.749	4.183	3.914	4.901	2.005
Maximum	6.488	6.307	6.083	5.074	6.079	7.110	2.981
Minimum	1.973	1.428	-0.218	0.973	0.421	1.920	0.141
Std. Dev.	1.087	1.361	1.678	1.113	1.445	1.151	0.670
Skewness	0.072	0.145	-0.293	-0.778	-0.388	-0.490	-0.846
Kurtosis	1.884	1.727	1.787	2.199	2.041	2.408	2.561
Jarque-Bera	12.818	17.253	18.386	30.996	15.388	13.262	30.922
Probability	0.002	0.000	0.000	0.000	0.000	0.001	0.000

In this table, we report the summary statistic of the 2YBYs and 30YBYs in panel A and panel B, respectively

**Fig. 1:**
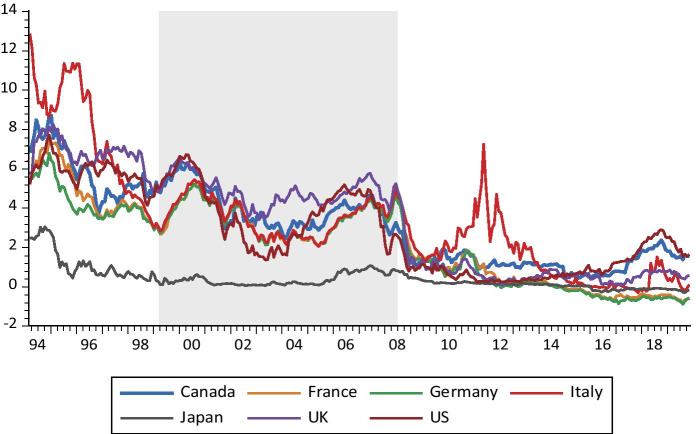
The dynamics of two-year sovereign bond yields

**Fig. 2:**
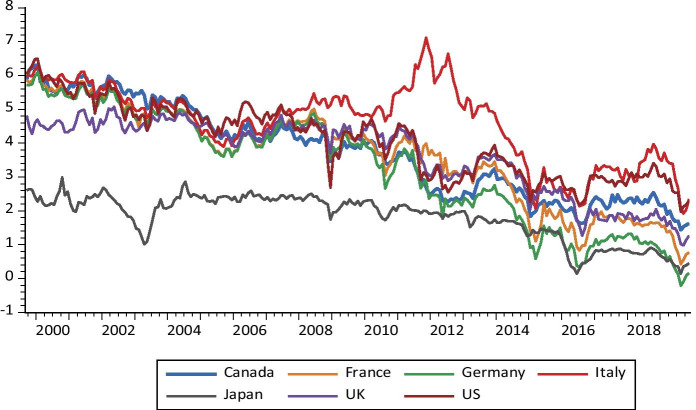
The dynamics of thirty-year sovereign bond yields

Figures 1 and 2 depict the dynamic of 2YBYs and 30YBYs, respectively. Both figures indicate that the series of SBYs evolved. All 2YBY and 30YBY series exhibited a downward trend. More significantly, the SBYs co-move throughout the studied period. This joint dynamic of bond yields motivated the investigation of the degree and patterns of connectedness between them. Figure 1 shows clearly the presence of three regimes in the dynamic of the 2YBYs along the considered period. The first covers the period before 1999_M03_, before the adoption of the euro as the European Union’s official currency. The second, from 1999_M04_ to 2008_M06_, which corresponds to the period before the 2007 GFC. The third covers the post 2008_M07_ period, which corresponds to the period after the GFC. Figure 2 indicates that there were no specific regimes detected for 30YBYs.

## Methodology

### Bond yield connectedness

The methodological framework of this empirical study aimed to construct connectedness measures following the methodology developed by Diebold and Yilmaz (2012). The dynamic total and net connectedness series among SBYs were extracted, and several panel data models were used to identify the drivers of the degree of connectedness between these bond yields. As advocated by the authors, an effective way to assess the degree of connectedness across different financial assets in the time domain is to consider a vector autoregressive ($VAR$) process and evaluate its forecast error variance decomposition. Formally, a $VAR$ model with $n$ variables and $p$ lags is written as

1 $$X_t={\textstyle\sum_{i=1}^p}\Phi_iX_{t-1}+\epsilon_t$$

where ${X}_{t}$ is the $n\times n$ autoregressive coefficient matrices, and ${\epsilon }_{t}$ represents the error term with zero mean and covariance matrix $\Sigma$. Under the condition of the covariance stationary, the moving average representation of the Eq. (1) is given as ${X}_{t}=\sum_{j=1}^{\infty }{A}_{j}{\epsilon }_{t}$, where ${A}_{j}$ is an $n\times n$ coefficient matrix that can be computed recursively following the form ${A}_{j}={\Phi }_{1}{A}_{j-1}+{\Phi }_{2}{A}_{j-2}+$ … ${\Phi }_{p}{A}_{j-p}$, with ${A}_{0}$, an $n\times n$ identity matrix and ${A}_{j}=0$ for $j<0$. The transformations, such as the variance decomposition and impulse response functions, are the key to understanding the dynamics of the system. Strictly, the variance decompositions allow us to assess the fraction of the *H*-step-ahead error variance in forecasting ${X}_{i}$ that is due to shocks to ${X}_{j}$ , $\forall j\ne i$ for each $i$.

The generalised forecast-error variance decompositions of the moving average representation of the *VAR* model allows generating the total, the directional and net spillovers.

The H-step-ahead generalised forecast-error variance decomposition as proposed by Koop et al. (1996) and Pesaran and Shin (1998) is

2 $${\theta }_{ij}^{g}\left(H\right)=\frac{{\sigma }_{jj}^{-1}{\sum }_{h=0}^{H-1}{\left({e}'_{i}{A}_{h}\Sigma {e}_{j}\right)}^{2}}{{\sum }_{h=0}^{H-1}\left({e}'_{i}{A}_{h}\Sigma {{A}_{h}^{^{\prime}}e}_{i}\right)}$$

where $\Sigma$ is the estimated variance matrix of the error vector $\varepsilon$ and ${\sigma }_{jj}$ is the standard deviation of the error term of the ${j}^{th}$ equation. In this equation, ${e}_{i}$ is a selection vector with a value of 1 for the ${i}^{th}$ element and zero otherwise. The normalise KPPS *H*-step-ahead forecast error variance decompositions can be expressed as:

3 $$\tilde\theta_{ij}^g\left(H\right)=\frac{\theta_{ij}^g\left(H\right)}{\sum_{j=1}^N\theta_{ij}^g\left(H\right)}$$

Using the volatility contributions from the normalised *H*-step-ahead forecast error variance, Diebold and Yilmaz (2012) proposed different measures that allow the description of the different patterns of spillovers or connectedness. Table 2 reports the different used measures.

**Table 2:** Measures of spillovers and connectedness

Measure	Index	Description
Total connectedness	$$S^g\left(H\right)=\frac{\sum_{\begin{array}{c}i,j=1\\i\neq j\end{array}}^N\tilde\theta_{ij}^g(H)}N\times100$$	This equation evaluates the contribution of spillovers of volatility shocks across sovereign bonds to the total forecast error variance.
Directional connectedness $i\leftarrow j$	$$S_{i.}^g\left(H\right)=\frac{\sum_{\begin{array}{c}i,j=1\\i\neq j\end{array}}^N\tilde\theta_{ij}^g(H)}N\times100$$	This equation measures the directional volatility spillovers received by sovereign bond $i$ from all other sovereign bonds $j$.
Directional connectedness $i\longrightarrow j$	$$S_{.i}^g\left(H\right)=\frac{\sum_{\begin{array}{c}i,j=1\\i\neq j\end{array}}^N\tilde\theta_{ji}^g(H)}N\times100$$	This equation measures the directional volatility spillovers transmitted by sovereign bond $i$ to all other sovereign bonds $j$.
Net connectedness	${S}_{i}^{g}\left(H\right)={S}_{.i}^{g}\left(H\right)-{S}_{i.}^{jg}\left(H\right)$	Measures the net volatility spillover from sovereign bond $i$ to all other sovereign bond $j$.
Net pairwise volatility connectedness	$S_{ij}^g\left(H\right)=\left(\frac{\tilde\theta_{ji}^g\left(H\right)-\tilde\theta_{ij}^g\left(H\right)}N\right)\times100$	Measures the difference between the gross volatility shocks transmitted from sovereign bond $i$ to sovereign bond *j* and those transmitted from *j* to *i*.

### Determinants of dynamic connectedness

To determine the factors influencing the directional connectedness among SBYs of selected developed countries, we estimate several panel data models. A close appraisal of existing literature suggests that macroeconomic factors offer the primary explanation for spillovers in bond markets. Following Claeys and Vašíček (2014) and Benlagha and Hemrit (2020), we considered inflation represented by the CPI and the real interest rate.

In this paper, to extend the models developed in the existing literature, we added the specific EPU index of each country as a potential contributing factor of the directional connectedness among SBYs. The general specification of the empirical model is expressed as

4 $${DC}_{it}={\beta }_{0}+{\beta }_{1}{EPU}_{it}+{\beta }_{2}{CPI}_{it}{+{\beta }_{3}INTR}_{it}+{\eta }_{i}+{\gamma }_{t}+{\varepsilon }_{it}$$

where $$\eta$$ is the time-invariant country-specific effect, $$\gamma$$ is the country-invariant time-specific effect and $${\varepsilon }_{it}$$ represents the idiosyncratic error. This error differs across individuals and evolves over time. In Eq. (4), the variable of interest (dependent) is the directional connectedness presented by the dynamic net connectedness between SBYs. The explanatory variables are the economic policy uncertainty index ($${EPU}_{it})$$, the inflation measured by the consumer price index ($${CPI}_{it}$$) and real interest rate ($${INTR}_{it}$$).

## Empirical results

### Unconditional patterns

Tables 3 and 4 are the volatility connectedness tables for 2YBYs and 30 YBYs, respectively. The results show that the net connectedness elements are similar for both bond markets composed of 2YBYs and 30 YBYs for the G7 countries. Therefore, being a transmitter or recipient of connectedness seems to be independent of the maturity period of the sovereign bonds. However, the total connectedness between 30 YBYs is significantly higher that of the 2YBYs with values of 69.70 and 57.81 indicating that, on average, across the whole sample of 30 YBYs (2YBYs), 69.70% (57.81) of the volatility forecast error variance in all seven countries comes from spillovers. In contrast with the net connectedness patterns, the total connectedness measures vary with the maturity period of the sovereign bonds. Moreover, is important to note that for both sovereign bond in the G7 countries the total connectedness are high enough to conclude that the larger part of the volatility forecast error variance comes from spillovers.

**Table 3:** Total dynamic connectedness among the 2YBYs

	US	Canada	UK	Germany	France	Italy	Japan	FROM
US	43.265	12.859	23.328	9.021	7.827	2.149	1.551	56.735
Canada	24.540	32.377	18.027	11.746	9.695	2.000	1.615	67.623
UK	18.018	12.818	44.599	13.120	8.979	0.775	1.691	55.401
Germany	16.873	10.928	18.008	27.612	21.611	1.809	3.160	72.388
France	16.288	10.671	16.257	26.015	23.808	3.653	3.308	76.192
Italy	9.701	8.032	3.680	9.569	14.099	53.112	1.807	46.888
Japan	6.448	2.879	5.048	6.201	5.729	3.186	70.510	29.490
CTO	91.867	58.187	84.347	75.672	67.939	13.573	13.132	404.718
CIO	135.132	90.565	128.946	103.284	91.747	66.685	83.642	TCI
Net connectedness	35.132	-9.435	28.946	3.284	-8.253	-33.315	-16.358	57.817

*CTO* Contribution TO others, *CIO* Contribution including own

**Table 4:** Total dynamic connectedness among the 30YBYs

	US	Canada	UK	Germany	France	Italy	Japan	FROM
US	24.729	18.723	13.246	18.178	14.344	2.645	8.133	75.271
Canada	17.749	26.497	12.023	19.053	15.443	3.488	5.749	73.503
UK	13.247	14.067	24.333	20.807	15.958	3.969	7.619	75.667
Germany	13.290	13.549	12.045	27.103	21.072	6.999	5.941	72.897
France	12.561	12.610	11.271	23.993	22.285	12.902	4.377	77.715
Italy	8.042	6.800	9.241	14.196	16.273	44.789	0.658	55.211
Japan	12.279	8.187	10.706	14.091	9.292	3.146	42.299	57.701
CTO	77.168	73.938	68.533	110.318	92.383	33.149	32.477	487.964
CIO	101.897	100.434	92.866	137.421	114.668	77.938	74.776	TCI
Net connectedness	1.897	0.434	-7.134	37.421	14.668	-22.062	-25.224	69.709

*CTO* Contribution TO others, *CIO* Contribution including own

To explore in more depth the connectedness behaviour of the considered bond yields we focus our analysis on the 2YBYs connectedness results reported in Table 3.

Table 3 shows that net connectedness elements between the 2YBYs of Canada, France, Italy and Japan are net recipients of connectedness, whereas the 2YBYs of US, UK and Germany are transmitters of connectedness effects. The off-diagonal entries in the US row – the relative influence of cross-variable shocks on US bond yields – by corollary must add up to 56.7 %, as revealed in the closing column of Table 3. The UK, at 14.2%, is the highest other-country contributor to the US’s decomposition. It also seems that SBYs in France react more strongly to the sovereign bond market in Germany (26%). Italy, at 0.77%, had the least influence among the G7 markets on the UK’s sovereign bond market over the sample period. The last row of the Table 3 indicates that sovereign bond markets in Japan and Italy showed relatively low levels of cumulative influence on others (at 13.13% and 13.57%, respectively). In summary, Table 3 displays Japan and Italy’s spillover from and to other countries being relatively low in general over the entire period and, at the country level, they are having their strongest interactions with France and US, respectively^1^.

### Conditioning and dynamics

**Fig. 3:**
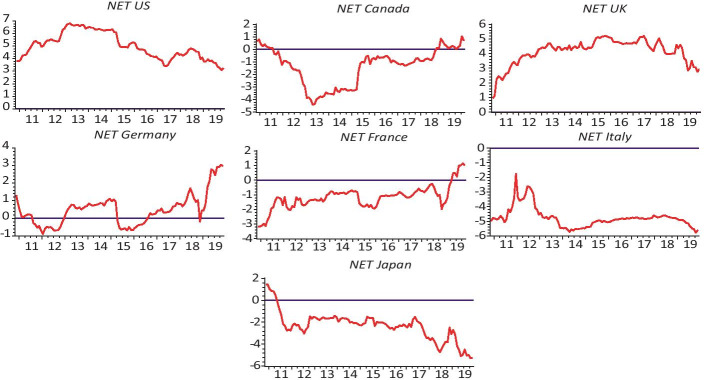
Net connectedness among the 2YBYs for every country in our sample

**Fig. 4:**
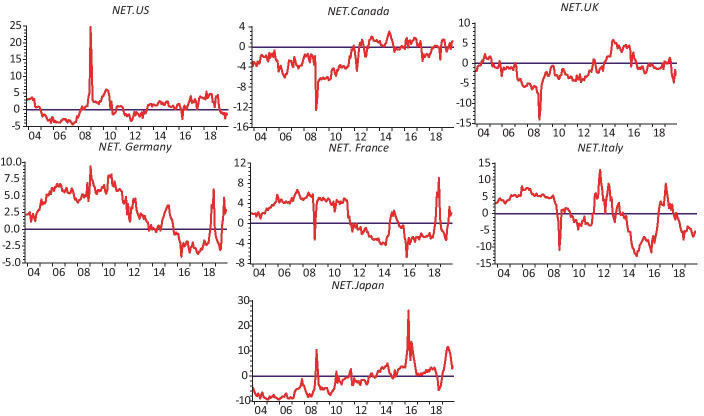
Net connectedness among the 30YBYs for every country in our sample

In order to explore the direction of net connectedness and, hence, the dynamic association among sovereign bonds, we used the dynamic spillover methodology proposed by Diebold and Yilmaz (2012). Figures 3 and 4 display the dynamic net connectedness indexes among 2YBYs and 30YBYs, respectively.

Figure 3 indicates that the US bond market is, mostly, a transmitter of the connectedness of 2YBYs. The dynamic net connectedness and the presence of several peaks in US sovereign bond yields are due to several events. First, following the terror attacks of September 11, 2001, the NC of the US increased more than 20%. Second, the US subprime mortgage crisis in 2007 occurred between 2007 and 2010 and resulting to the Lehman Brothers’ bankruptcy that occured in September 2008, the net connectedness of the US sovereign bond increased to 10% and did not come down until the third quarter of 2011. Moreover, due to the worsening of sovereign debt and banking crisis that happened in 2011, the net connectedness decreased, and the sovereign bond market becomes the receiver of volatility shocks. Again, the net connectedness of the US sovereign bonds market jumped in 2016, when West Texas Intermediate oil prices collapsed. This collapse is explained by the fact that the US sovereign bonds market exports more than it imports which strengthen productive capacity to achieve balanced, non-inflationary growth, but the crisis with first appeared in the US can spread in a broader in the near term.

Moreover, the increasing manifestation of foreign investors who have intensified cross-border relationships makes the US sovereign bond market the leader of the global financial market. Sovereign bond volatility can quickly propagate the US economy’s stress to other countries. Apart from the US sovereign bond market, it was found that the US sovereign bond markets radiate fragile mean spillovers to Canada because the Canadian sovereign bond market has experienced significant negative net connectedness on several occasions. Further, the cross-volatility spillover factor from US sovereign bond markets to its analogue in Canada was found to be noteworthy. The fact hidden behind this result may be that sovereign bond market in Canada is affected negatively by its closeness to the US, where strong economic agreements and companies’ interactions turn these two North American countries into a neighbouring economy.

The net connectedness of the UK sovereign bond market lay under zero for a most of the sample period, which suggests that this country was receiving volatility shocks transmitted from other sovereign bond markets. Negative net connectedness occurred throughout the studied period, except in three cases: 1) during the British military intervention in Iraq that began in 2003 and the subsequent oil revenues crash; 2) during the American subprime mortgage crisis; and 3) throughout the debates about pro-Brexit and pro-EU starting in January 2013. The other three European countries, namely, Germany, France and Italy exhibit diverse net connectedness patterns. Earlier to the liquidity crunch that occurred in 2007, the directional connectedness measures of Germany, France and Italy sovereign bonds volatility show somewhat similar level, path and pattern. Thus, the net pairwise connectedness among various combinations of markets with these countries is the lowest.

Our empirical results show that, following the subprime crisis, these countries display several distinctive features that indicate that the European countries were sensitive to external shocks. Succeeding the Greek debt crisis that occurred in 2010, the net connectedness of German sovereign bond market with other sovereign bond markets became positive. Moreover, the dynamic connectedness shows that the net transmitter of shocks in Germany grew slightly larger than the net receiver of shocks since the sharp fall in oil prices in June 2014. The France sovereign bond market had been a receiver of volatility spillovers since the ESDC that began in 2008. However, during the 2017–2019 GFC, France’s net connectedness was negative. Because Italy was dramatically affected by the sovereign debt crisis in 2017 leading to higher connectedness to others It is worth mentioning here that, although these three European countries irregularly had negative values of net connectedness, their values were small, generally below 12%.

Overall, according to our findings, an upsurge of the volatility of the foremost markets was conveyed in fairly different ways to the European sovereign bond markets. Similar features can be observed for Japan in that it has been a net recipient of the volatility of sovereign bonds from other countries in most periods. However, between 2005 and 2009, Japan affected the behaviour of the other sovereign bond markets in the G7 to a great extent. The net connectedness was highly volatile during the great global recession when the economy was shocked by the Great East Japan Earthquake and resulting Tsunami; it shows sharp jumps that exceed the 30% mark during this period. Its net connectedness increased a minimum of nearly 40% during the period from 2013 to 2016. This finding may advocate that the effect of Japan has progressively increased with the simulative monetary and fiscal policies that went into effect in 2013, which were probably the result of the long-lasting economic crisis that Japan confronted.

Regarding the results of net connectedness between sovereign thirty-year bond markets, Fig. 4 highlights two major findings. First, the levels of bond yields and net connectedness are large and exhibit some asymmetric patterns. Second, net directional connectedness in US sovereign bond market is small and insignificant, while the connectdness effects are significant in other countries. It should be mentioned here that, during the first break of the subprime crisis period, the net connectedness of the US amplified to 28% but declined immediately after. For the sovereign bond market in Canada, the plots show that this market, similarly, looks to be the main receiver of volatility spillover shocks during the whole studied period. After a brief break in 2013 due to a broad-based decline in the Toronto Stock Exchange in June 2013, the net connectedness became predominantly stable. For most of the period before 2014, the UK sovereign bond market was the net receiver of shocks from other sovereign bond markets shocks and reached its global maximum (Almost 14%) during the GFC period. Aside from other European markets, we observed significant directional spillover return predictability between some sovereign bond markets.

The results show that Germany was at the transmitting end of the net connectedness in most of the time over the entire baseline sample period until 2016, following a series of news disclosing Deutsche Bank’s troubled financial position. One day after the Brexit referendum in June 2016, Germany became a net receiver of volatility shocks from others. Before the GFC, we find that Italy and France’s sovereign bond markets were the net transmitters of volatility spillovers shock to other markets, implying that these markets are the foremost drivers of the bond market volatility of other G7 countries. After 2011, the Lehman collapse, the Greek bailout and the Cyprus bailout were among the main factors that strengthened the contagion in these countries and, generally, led to a sharp increase of the magnitude of connectedness across markets.

Japan was also at the receiving end of net connectedness – similar to the net connectedness observed among the 2YBYs – with a lower magnitude of volatility spillover. It appears to have been largely a net receiver of volatility shocks from others, although the pattern and magnitude of shock spillovers were more pronounced in the first sample period. Its net connectedness had trended strongly upward, which is visible only in 2009 and 2016 during which it reached 11% and 29%, respectively. These figures indicate that Japan still follows its cousin in the US following the housing boom and bust in 2007 to 2009. Our results are consistent with the existing literature that provides evidence supporting the increase of spillovers and connectedness among sovereign bond markets (De Santis and Zimic 2018; Ahmad et al. 2018; Antonakakis and Vergos 2013). The market linkages became stronger in the crisis periods. Moreover, our findings highlight that the developed sovereign markets in the US, Japan and Germany, in general, tend to be the source of contagious spillover, while the UK and Canadian markets tend to be recipients of such spillover. Furthermore, the direction and intensity of net connectedness across sovereign bond markets are sensitive to financial and economic events.

The identification of the underlying determinants of net connectedness is important, not only for causing issues such as the home bias in sovereign bond holdings (Lane 2005, 2012) but also for practical concerns such as the development of proper financial market monitoring measures. Behind all the different reasons for the upward and downward revision of net connectedness, there is one common factor: rising uncertainty. It quickly became evident that uncertainty over economic policy plays a key role in economic outcomes over time. Thus, being able to identify the main determinants of forecasting the increased net connectedness among sovereign bonds markets with a maturity of two and thirty years could help public firms, international portfolio holders and government policymakers to be better prepared for and perhaps take steps to redress some of the effects of net connectedness in the short and long terms. To accurately measure the underlying factors behind net connectedness, it is necessary to understand the effect of EPU and the components of some economic characteristics on the connectedness between 2YBYs and 30YBYs.

### The determinants of the connectedness between 2YBYs and 30YBYs

This section discusses the panel results where the dynamic connectedness between sovereign 2YBYs and 30YBYs is the dependent variable. Columns 2 to 4 are from the 2YBY sample, columns 5 to 7 are from the 30YBY sample. In the specification, we include some general macroeconomic variables, especially inflation CPI and interest rate (INTR), and EPU, in order to confirm whether the uncertainty keeps its forecasting power when controlling for the other macroeconomic measures. Standard errors are robust to heteroscedasticity and are clustered at the country-pair level. Different econometric estimation techniques are used to check for robustness: the pooled OLS, the fixed effect and the random effect.

According to Table 5, all the estimated models provide the expected signs and for significant coefficients of the economic variables. Moreover, the estimated coefficients are and vary slightly from model to another. For the pooled OLS model, all individually specific effects were completely ignored, because basic assumptions such as the orthogonality of the error term were violated. Moreover, as an illustration, the random effect estimator was not suitable to the used data since the null hypothesis of significant random effects was rejected by the Hausman test for *p-value* equal to zero. Therefore, we relied mostly on the fixed effect for the interpretation of regression results.

**Table 5:** The determinants of the net connectedness among 2YBYs and 30YBYs

	2 years bond yields				30 years bond yields		
	Pooled OLS	Fixed Effect	Random Effect		Pooled OLS	Fixed Effect	Random Effect
Intercept	-1.515^**^	15.85^***^	5.347^***^		-1.399^**^	-7.742^***^	-5.116^**^
	(0.518)	(1.843)	(1.445)		(0.477)	(2.004)	(1.768)
EPU	-0.0032^**^	0.00395^**^	0.00360^**^		0.00184	0.000612	0.000976
	(0.0019)	(0.00202)	(0.00205)		(0.00172)	(0.00186)	(0.00185)
CPI	0.0231^***^	-0.130^***^	-0.0476^***^		0.00531^*^	0.0525^**^	0.0313^*^
	(0.0028)	(0.0139)	(0.00973)		(0.00255)	(0.0160)	(0.0122)
INTR	-0.210^**^	-0.734^***^	-0.213*		0.221^**^	0.695^***^	0.585^***^
	(0.0786)	(0.132)	(0.115)		(0.0812)	(0.127)	(0.116)
*N*	1659	1659	1659		1316	1316	1316
*R*^2^	0.044	0.052	-		0.009	0.025	-
*rho*	-	.727	.093		-	.385	.252

This table presents the results from a panel data method say Pooled, fixed and Random effects models. We consider sovereign bonds with maturities of 2-years and 30 years. The time period is from January 2015 until December 2019. Standard errors in parentheses *rho* measures the fraction of variance due to *u*_*i*_

^*^ *p* < 0.1; ^**^ *p* < 0.05; ^***^ *p* < 0.01

Most importantly, for our purpose, the results show that policy uncertainty is significantly associated with the connectedness levels of 2YBYs. The positive influence of the policy uncertainty index on the net connectedness reveals that higher uncertainty in economic policy surges the investor perceptions of the global shocks in the shorter-term bonds associated with the substantial declines in market frictions such as trading costs, transaction fees and taxes, thus, leading to portfolio reallocation in search of risk-adjusted yields and international diversification opportunities.

According to the literature, which documents that herding is more likely to appear in periods of great uncertainty, and researchers, who have been sceptical about the rationality of investors’ decisions, investors are not fully rational because they tend to be influenced by uncertainty as a possible driver of the deviation of SBYs and bond prices from their fundamental values, thus leading to herding behaviour (Galariotis et al. 2015). This finding reveals strong evidence that confusing economic policy orientations from one country fully participate in intensifying the spillover effects across sovereign bond markets rising from the associated widening in individual countries’ sovereign spreads in the bond market. Co-movement in the international sovereign short-term bonds that generates spillover effects will reduce the benefit or even eliminate the possibility for global investors to benefit from international portfolio diversification and reduce the intertemporal global portfolio choices (Antonakakis et al. 2018). Overall, EPU makes investors flee the country and decreases investment and development in the short run as domestic sovereign bonds are unable to provide smoothing. In contrast, our analysis highlights no significant effect of policy uncertainty on thirty-year bond yield spillover, which implies that the influence of economic policy uncertainty almost disappears for larger investment horizons in bonds with thirty-year maturities. This relationship may be explained by policy uncertainty, which can cause a decrease in aggregate consumption and real economic activity; thus, returns increase in a global condition in which investors can profit from raising the additional interest rate income.

At the macroeconomic level, in the wake of heightened policy uncertainty, the government can still use taxes and spending to stabilise the economy in the long term, an essential prerequisite for the stability of expected inflation, expected real rates and the term premium (Claeys 2017). As a result, governments may achieve higher interest income domestically forces agents to invest in the national capital market without lending abroad. Thus, the effect of EPU in these economies would be imperceptible, and this could lead, through portfolio balance effects among financially interconnected economies, to a limitation or reduction in capital inflows and lower yields and a low-term premium of international sovereign bonds. Beyond the significance of the economic uncertainty variables considered in the model, it is important to determine whether macroeconomic indicators are statistically significant. It is apparent from our findings that the interest rate is effective as a monetary policy instrument in reducing (or raising) the connectedness between 2YBYs and 30YBYs.

Both researchers and policymakers acknowledge that, when the central banks need to raise rates in order to keep the economy from overheating (*contractionary monetary policy*), this has tremendous repercussions on market economies. This upsurge is expected to worsen budget balances and compromise a country’s ability to pay its debt, thus bringing the yields up and making sovereign bonds attractive from a return point of view. The rise of the SBY then leads to a sharp reduction in capital flows to other countries. Moreover, Belke and Verheyen (2014) suggest that the low-interest rate in advanced economies results in favourable liquidity conditions and has driven investors to foreign bonds in search of higher expected risk-adjusted returns. Consequently, interest rate reduction was favourable for supporting connectedness among 2YBYs in our sample countries. For connectedness between 30YBYs, our findings suggest that rising interest rates – whether stemming from conventional policy adjustments, forward guidance, or other forms of signalling – have been positive effects on this connectedness among bond yields for several countries. Thus, the interest rate shocks can affect sovereign bond prices globally and the business cycles across countries which can distinctly diverge.

The impact of inflation has a significant coefficient and expected sign for two-year bond market co-movement. This result can be interpreted as an evident effect: positive short-run changes in inflation over expectations result in a temporary rise of bond yields, so investors will demand a higher yield to compensate for inflation risk. This finding agrees with recent studies (Poghosyan 2014; Yusuf and Prasetyo 2019). According to Albagli et al. (2019), and such a response could be motivated by inflationary pressures from exchange rate pass-through and trade balance considerations. Conversely, it is noteworthy that this effect is statistically positive for thirty-year bond market co-movements with a much lower level of significance, which confirms the results of Chionis et al. (2014). Poghosyan (2014) suggest that SBYs can provisionally deviate from their long-run equilibrium level driven by short-run factors, such as inflation and other monetary policies.

## Conclusion and policy implications

Our paper examined two major issues related to (i) the drawing of a complete picture of the connectedness between various sovereign two- and thirty-year bond yields among the considered markets and (ii) to the effect of the economic policy uncertainty EPU and related macroeconomic variables as the inflation, the interest rate on the net connectedness patterns.

There are three conclusions from our study. First, the total volatility connectedness across the G7 countries are significantly high for both two year and three year sovereign bond yields. However, the total connectedness increases with the time horizon of the sovereign bonds.

Second, the patterns of the dynamic connectedness varies among the G7 countries and with the time horizon of the considered sovereign bonds. These variations are mainly related to several economic and political shocks, such as terror attacks of September 11, 2001, the US subprime mortgage crisis for the US, Brexit referendum in June 2016 for UK and the Greek bailout for the European countries.

Third, we have shown that uncertainty about economic policy has had a positive effect on the net connectedness of 2YBYs, but it has no statistically significant effect on that of 30YBYs. The empirical evidence generally settles that EPU can elicit significant reactions from the sovereign bond markets in the short-term between various financial markets, given the amplification of the biases of individual investors to higher levels of extreme behaviour.

The findings of this study have several implications for investors and portfolio managers. Since the total connectedness increases with the time horizon of the sovereign bonds, investors in international markets are suggested to form a diversified portfolio composed of sovereign bond with different maturity dates. Moreover, investors should pay attention to the increased political economic uncertainty in the countries issuing the sovereign bonds in which they are willing to invest.

## Footnotes

1 To save space we have reported the analysis of the results of Table 4 in the supplementary documents since the analysis is quite similar to that for Table 3.
